# Metabolomic Profiles Are Gender, Disease and Time Specific in the Interleukin-10 Gene-Deficient Mouse Model of Inflammatory Bowel Disease

**DOI:** 10.1371/journal.pone.0067654

**Published:** 2013-07-09

**Authors:** Victor K. Tso, Beate C. Sydora, Rae R. Foshaug, Thomas A. Churchill, Jason Doyle, Carolyn M. Slupsky, Richard N. Fedorak

**Affiliations:** 1 Centre of Excellence for Gastrointestinal Inflammation and Immunity Research, Division of Gastroenterology, Department of Medicine, University of Alberta, Edmonton, Alberta, Canada; 2 Department of Surgery, University of Alberta, Edmonton, Alberta, Canada; 3 Department of Laboratory Medicine and Pathology, Vernon Jubilee Hospital, Vernon, British Columbia, Canada; 4 Department of Nutrition, University of California Davis, Davis, California, United States of America; Institut Pasteur de Lille, France

## Abstract

Metabolomic profiling can be used to study disease-induced changes in inflammatory bowel diseases (IBD). The aim of this study was to investigate the difference in the metabolomic profile of males and females as they developed IBD. Using the IL-10 gene-deficient mouse model of IBD and wild-type mice, urine at age 4, 6, 8, 12, 16, and 20 weeks was collected and analyzed by nuclear magnetic resonance (NMR) spectroscopy. Multivariate data analysis was employed to assess differences in metabolomic profiles that occurred as a consequence of IBD development and severity (at week 20). These changes were contrasted to those that occurred as a consequence of gender. Our results demonstrate that both IL-10 gene-deficient and wild-type mice exhibit gender-related changes in urinary metabolomic profile over time. Some male-female separating metabolites are common to both IL-10 gene-deficient and control wild-type mice and, therefore, appear to be related predominantly to gender maturation. In addition, we were able to identify gender-separating metabolites that are unique for IL-10 gene-deficient and wild-type mice and, therefore, may be indicative of a gender-specific involvement in the development and severity of the intestinal inflammation. The comparison of the gender-separating metabolomic profile from IL-10 gene-deficient mice and wild-type mice during the development of IBD allowed us to identify changes in profile patterns that appear to be imperative in the development of intestinal inflammation, but yet central to gender-related differences in IBD development. The knowledge of metabolomic profile differences by gender and by disease severity has potential clinical implications in the design of both biomarkers of disease as well as the development of optimal therapies.

## Introduction

Metabolic profiling can be used to study normal variations as well as disease-induced changes in cellular and molecular mechanisms. The comparison of metabolic profiles from various bio-fluids is a powerful tool to investigate physiological or pathophysiological changes under defined conditions, such as gender and nutritional intake, at any given time point [1], [2]. Furthermore, metabolomic analysis can identify metabolites with potential importance as biomarkers in discriminating between normal and pathological conditions.

Inflammatory bowel diseases (IBD), including Crohn’s disease (CD) and ulcerative colitis (UC), are chronic, intestinal diseases of unknown etiology [3]. Characteristics of these diseases are uncontrolled immune responses to components of the endogenous micro biota resulting in imbalances in cytokine production and severe intestinal inflammation [4]–[6]. Recently, metabolomics has been introduced to study pathophysiological differences in IBD in humans [7]–[10] as well as in rodent models [11]–[16]. The metabolomic changes accompanying the inflammatory response led to distinct profiles that can be used to differentiate IBD from non-IBD.

Treating women with IBD encounters different challenges than treating men with IBD [17]. Furthermore, IBD-related gender differences have been reported with regard to disease activity [18], cardiovascular complications [19], urinary tract infections [20], along with reduced deoxyribonuclease I serum activity levels, which appears to have pathogenic consequences [21]. Conversely, other studies have shown that gender does not appear to be a major contributing factor in IBD [22]. The contradiction of these studies helps to highlight our lack of understanding regarding the role gender plays in IBD.

Gender differences have also been reported for other inflammatory disorders, including cardiovascular diseases, hepatocellular carcinoma, diabetes, non-alcoholic fatty liver disease, osteoporosis, and autoimmune diseases [23]–[27]. Largely, these differences are attributed to gender-specific influences on immunological responses [28], [29]. In addition, gender-specific hormones themselves can impact disease development and severity [30], [31]. Gender differences are also well documented with regard to pharmacokinetics, manifested in drug toxicity and therapeutic responses, and transport of endogenous and exogenous compounds in a variety of organs [32]. Healthy male and female metabolism also differs when assessed under similar conditions. This has been studied primarily in regard to fat and carbohydrate metabolism in the context of exercise and obesity [33], [34]. For example, during exercise, females oxidize proportionally more lipids and less carbohydrate than males [35], [36], likely as a consequence of higher estrogen concentrations [37]. Basal fat oxidation is lower in females than in males [33] resulting in proportionally higher body fat, again, likely due to the effect of sex steroids and differences in insulin resistance [34].

Gender differences have been investigated by RNA and microarray analysis leading to the identification of distinct metabolites. Recently, NMR metabolomics has been employed to study male versus female differences [38], and analysis of urine and plasma has resulted in clear gender separations in principal component analysis (PCA) profiles [1].

To date, the metabolite differences generated by gender and the resulting metabolomic profile have not been examined in IBD. A detailed understanding of gender-related metabolite differences will be important in the development of metabolomic biomarkers for diagnosis and response to therapy. Using a mouse model of IBD, we investigated differences in the metabolomic profile between males and females as they progressed through onset of intestinal inflammation, and compared these changes to healthy control mice without intestinal inflammation.

## Materials and Methods

### Mice

IL-10 gene-deficient mice on 129Sv/Ev background and control wild-type 129Sv/Ev mice were housed under specific pathogen-free (SPF) conditions at the University of Alberta’s animal facility. Mice were weaned at 4 weeks of age, which coincided with the first urine collection time point. Urine was collected thereafter at week 6, 8, 12, 16, and 20. Mice were sacrificed at week 20 for histopathologic analysis and assessment of cytokine secretion. Ten mice were used in each group.

### Ethics Statement

This study was carried out in strict accordance with the recommendations in the Canadian Council for Animal Care. The protocol was approved by the Committee on Animal Ethics at the University of Alberta.

### Urine Collection in Metabolic Cages

For urine collection, mice were placed individually in metabolic cages for 22 hours with access to water, but without food. Both, fasting-induced stress responses as well as food intake can result in metabolic changes. Controlling the amount and time of food intake during the 22 hour urine collection period would have posed an additional challenge, especially in light of the fact that we were dealing with mice at various ages and gender and, thus, varying body weights and dietary requirements. Therefore, we opted for fasting during urine collection to keep external influences at a minimum. Urine was funneled into vials prepared with 100 µL paraffin oil to avoid loss from evaporation. Funnels were rinsed with 1.5 ml sterile water and an additional 500 µL solution containing 0.02% sodium azide (NaN3) before samples were harvested from collection vials and frozen at −80°C.

### Sample Preparation and NMR Acquisition

For nuclear magnetic resonance (NMR) acquisition, samples were thawed and debris was removed by filtering through a thoroughly cleaned Nanosep Omega 3 K spin filter (Pal Corporation). An aliquot of 585 µL was transferred to a clean vial containing 65 µL of 2,2-Dimethyl-2-silapentane-5-sulfonate-d6 sodium salt (DSS-d6) Chenomx internal standard (5.1436 mM DSS-d6, 0.196% NaN3 w/v, 98% D20 v/v) purchased from Chenomx Inc (Edmonton, AB, Canada). The pH was adjusted to pH 6.7–6.8 using HCl and NaOH buffers and measured using an Accumet XL 15 pH meter (Fisher Scientific, Canada). A 600 µl sample was then loaded into an NMR tube and inserted into the spectrometer.

NMR acquisition was performed on an Oxford 600 MHz NMR spectrometer with a Varian VNMRS two-channel console running VNMRJ software version 2.2C on a RHEL 4 host computer. The spectrometer was equipped with an HX probe with Z-axis gradients. Samples were run at a sweep width (sw) of 7225.43 Hz. The saturation frequency (sfrq), transmitter offset (tof), and pulse width (pw) were all individually calibrated at the start of each day. The tof typically ranged from −213 to −215 Hz, and the pw ranged from 6 to 8 µs. Shims were optimized until an acceptable line-width value was obtained at relative peak heights of: 50% (<1.0 Hz), 0.55% (<12.0 Hz), and 0.11% (<20.0 Hz). Finally, during post-processing, zero filling was used to resolve the actual acquired data points to the next higher factor of 2, and no weighting functions were applied.

We utilized the first increment of a 2D-^1^H,^1^H-NOESY pulse sequence for the acquisition of ^1^H-NMR data and for suppressing the solvent signal. Experiments used a 100 ms mixing time along with a 990 ms pre-saturation (∼80 Hz gammaB_1_). Spectra were collected at 25°C through a total of 32 scans over a period of 3.5 minutes using a total recycle delay of 5 seconds (i.e., a 1-second recovery delay/saturation and a 4-second acquisition).

### Spectral Analysis

Samples were processed to fine-tune the phasing of spectra as well as to eliminate the water peak and correct the baseline. The line shape of the spectra was set to a width of 1.3 Hz. Spectral analysis was done using Chenomx Inc NMR-suite software version 4.6. The chemical compound library was constructed for a pH ranging between 6 and 8. 2,2-Dimethyl-2-silapentane-5-sulfonate-d6 sodium salt (DSS-d6*)* was used as the internal standard. A total of 56 metabolites were identified and quantified, not including the DSS-d6 standard. After all spectra were analyzed, their metabolite concentrations were exported from the program into an Excel file.

### Statistical Analysis

The Excel files generated after spectral analysis were grouped according to strain, gender, and age and multivariate statistical analysis (principal Component Analysis (PCA) and partial least squares – discriminant Analysis (PLS-DA)) was performed using Simca P+ version 12.0.1 (Umetrics, Umea, Sweden).

### Analysis of Inflammation

Colons were excised from mice euthanized at 20 weeks of age. Tissues were cut longitudinally in half, washed in PBS, and fixed in 10% freshly prepared formalin. One half of each colon was then embedded in paraffin, cut, and stained with hematoxylin and eosin for light microscopy as previously described [39]. The slides were scored in a blinded fashion (JD) according to a scheme ranging from 0 (no injury) to 10 (maximal injury) as determined by four parameters: mucosal ulceration, epithelial hyperplasia, lamina propria mononuclear and neutrophilic infiltration [39]. The other half of the colon was weighed, placed in 1 mL RPMI culture medium, and incubated at 37°C, 5% CO_2_ in humidified atmosphere for 24 hours. IFNγ in the supernatants of these cultures were quantified by standard ELISA techniques using commercial IFNγ kits (R&D System Inc., Minneapolis, MN, USA). In order to normalize IFNγ release in individual cultures, the concentration of this cytokine in 1 mL culture was computed according to the weight of the tissue in this culture. Differences between mean values derived from groups of male and female mice were evaluated using *t*-tests or analysis of variance (Mann-Whitney Rank Sum Test), where appropriate (SigmaStat, Jandel Corporation, San Rafael, CA).

## Results

### Wild-type Male and Female Mice Cluster into Distinct Groups According to their Metabolomic Profile

**Figure 1:**
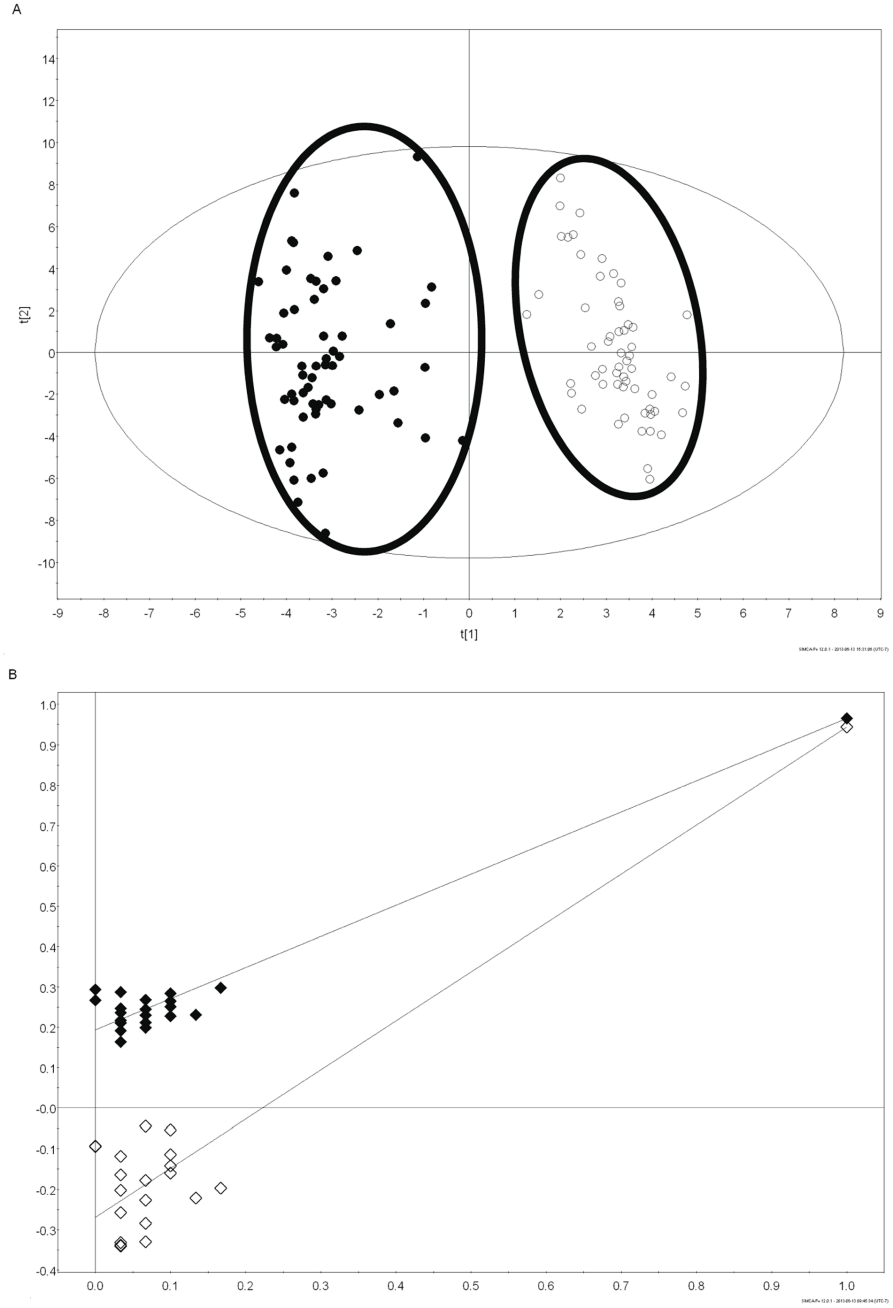
Metabolomic profiles of male and female wild-type mice. (A) Partial least squares-discriminant analysis (PLS-DA) of male (closed triangle) versus female (open triangle) wild-type mice over all time points tested at weeks 4, 6, 8, 12, 16, and 20 demonstrate a gender separation in two distinct clusters. (B) Validation of the PLS-DA plot using 20 random permutations of the data revealed an R^2^Y value of 0.965 and a Q^2^ value of 0.944, indicating that separation observed in the PLS-DA model in Figure 1A did not occur by chance. (R^2^Y = closed diamonds, Q^2^ = open diamonds).

Using healthy wild-type mice, we investigated differences between male and female mice using urine-based NMR metabolomic analysis. PLS-DA derived from metabolite concentrations in mouse urine collected at 4, 6, 8, 12, 16, and 20 weeks of age revealed that male and female mice separate into two distinct metabolomic clusters with no overlap (Figure 1A).

Validation by random permutation revealed positive slopes for both R^2^Y and Q^2^ with the original model having an R^2^Y of 0.965 and a Q^2^ of 0.944 (Figure 1B), indicating that separation observed in the PLS-DA model in Figure 1A did not occur by chance. In addition, no overlap was observed between metabolomic clusters of male and female mice when PLS-DA plots were graphed separately for the individual time points at 4, 6, 8, 12, 16, and 20 weeks of age. These graphs can be accessed as supplementary material (Figures S 1, S 2, S 3, S 4, S 5, S 6 in File S1).

To investigate which metabolites were responsible for the separation of male and female metabolomic clusters on the PLS-DA plots, we employed coefficient graphs examining 56 selected metabolites from the Chenomx Inc NMR-suite software. Metabolites with a variable importance in the projection (VIP) score of 1 and above were marked and identified in the graphs as those important for female differentiation (positive coefficient score) and male differentiation (negative coefficient score). This analysis revealed a total of 20 metabolites that directed the separation of wild-type females on the PLS-DA plots and 23 metabolites that directed the separation of wild-type males on the PLS-DA plots (Tables 1, 2).

**Table 1:** Gender specific metabolites that contribute to the separation of female wild-type control mice on the PLS-DA plot at different experimental time points.

Metabolites	Age (Weeks)					
	4	6	8	12	16	20
1. Formate	+	+	+		+	
2. Guanidoacetate	+				+	
3. Glycin	+					+
4. 1-Methylnicotinamide	+	+	+	+	+	+
5. Trimethylamin N-Oxide	+	+	+	+	+	+
6. 3-Methyl-2-Oxovalerate	+	+	+	+	+	+
7. Glutarate		+	+	+	+	
8. Lactate		+	+	+	+	+
9. Pyruvate		+	+		+	+
10. Glycolate		+	+		+	+
11. N-Carbamoyl-β-Alanine			+	+	+	+
12. 2-Oxoisocaproate			+	+	+	+
13. Acetate				+	+	
14. Fucose				+	+	+
15. Allantoin					+	+
16. Betaine					+	
17. cis-Aconitate					+	+
18. Mannose					+	+
19. 3-Indoxylsulfate						+
20. Phenylacetylglycine						+
**Total number of metabolites**	**6**	**8**	**10**	**9**	**17**	**15**

The plus sign (+) in a column indicates a metabolite that contributed to the separation of the respective gender on the PLS-DA plot.

**Table 2:** Gender specific metabolites that contribute to the separation of male wild-type control mice on the PLS-DA plot at different experimental time points.

Metabolites	Age (Weeks)					
	4	6	8	12	16	20
1. Uracil	+					
2. Trigonelline	+					
3. Dimethylamine	+					
4. N,N-Dimethylglycine	+	+	+			
5. 2-Hydroxyisobutyrate	+	+	+			
6. Isoleucine	+	+	+			
7. Adipate	+	+	+	+		
8. Citrate	+			+		
9. Ethanolamine	+	+		+	+	
10. Succinate	+	+	+	+	+	+
11. Trimethylamine	+	+	+	+	+	+
12. Methylamine	+	+	+	+	+	+
13. N-Isovaleroylglycine	+	+	+	+	+	+
14. Valine		+	+	+	+	+
15. Taurine		+	+	+	+	+
16. Butyrate		+	+	+		+
17. Creatine		+	+			
18. trans-Aconitate				+		
19. Pyroglutamate				+		
20. Methanol				+		
21. 3-Hydroxybutyrate				+		
22. Hippurate						+
23. 2-Aminobutyrate						+
**Total number of metabolites**	**13**	**13**	**12**	**14**	**7**	**9**

The plus sign (+) in a column indicates a metabolite that contributed to the separation of the respective gender on the PLS-DA plot.

We next examined which individual metabolites, contributing to the separation, were unique or similar to each gender. Three metabolites were unique to males at 4 weeks of age (uracil, trigonelline, and dimethylamine). Two metabolites were unique to females at 20 weeks of age (3-indoxylsulfate and phenylacetylglycine) and two unique to males at 20 weeks of age (2-aminobutyrate and hippurate). Metabolites that contributed to male and female separation of the PLS-DA plot at each time point included 1-methylnicotinamide, trimethylamine-N-oxide, and 3-methyl-2-oxovalerate for females and succinate, trimethylamine, methylamine, and N-isovaleroylglycine for males (Tables 1, 2). Other metabolites contribute to the separation on more than one but not on all time points, thus, creating a unique profile for male versus female wild-type mice.

### The Metabolomic Profile of IL-10 Gene-deficient Mice Cluster into Distinct Groups Relative to both Severity of IBD and Gender

Having shown that the metabolomic profiles separate due to gender in healthy wild-type control mice, we next established whether this gender-induced separation persisted, or was altered, when mice developed IBD.

**Figure 2:**
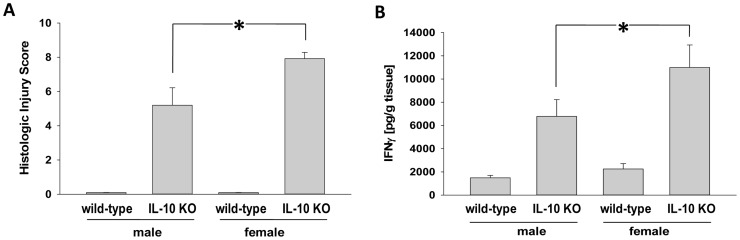
Histologic injury score and IFNγ immune response in male and female mice. (A) Histologic injury as determined by a scoring system ranging from 0 (no injury) to 10 (maximal injury). (B) IFNγ release in 24 hour colonic tissue culture as quantified by ELISA-based IFNγ kits. Graphs represent mean ± SE for n = 10 mice per group. *p<0.05.

Mice with a genetically-engineered deletion of the anti-inflammatory cytokine IL-10 develop an intestinal inflammation beginning at 8 weeks of age and reaches its peak sustained activity by 14 weeks of age [11], [40]. IL-10 gene-deficient mice were sacrificed at 20 weeks of age and characterized according to their intestinal histopathologic injury score as well as to their quantitative spontaneous inflammatory cytokine (IFNγ) release in cell culture. Intestinal inflammation was more severe in female mice; manifested in a significantly higher histologic injury score (Figure 2A), and significantly increased colonic IFNγ secretion (Figure 2B).

**Figure 3:**
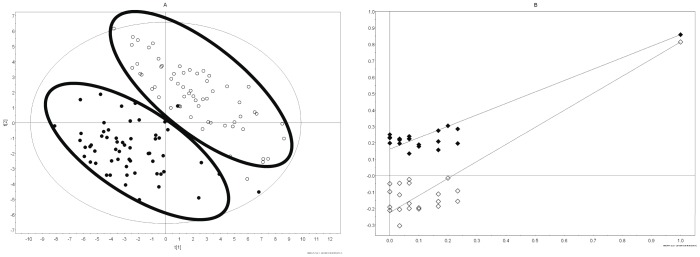
Comparison of metabolomic profiles between male and female IL-10 gene-deficient mice. (A) Partial least squares-discriminant analysis (PLS-DA) of male (closed circle) versus female (open circle) IL-10 gene-deficient mice over all time points tested at weeks 4, 6, 8, 12, 16, and 20 demonstrate a gender separation in two distinct clusters. (B) Validation of the PLS-DA plot using 20 random permutations of the data revealed an R^2^Y value of 0.858 and a Q^2^ value of 0.813, indicating that separation observed in the PLS-DA model in Figure 3A did not occur by chance. (R^2^Y = closed diamonds, Q^2^ = open diamonds.).

Similar to the results seen in the wild-type control mice (Figure 1), PLS-DA analysis derived from metabolite concentrations in IL-10 gene-deficient mouse urine collected at 4, 6, 8, 12, 16, and 20 weeks of age revealed that these male and female mice also separated into 2 distinct metabolomic clusters at each time point (Figure 3A). Validation by random permutation revealed positive slopes for both R^2^Y and Q^2^ with the original model having an R^2^Y of 0.858 and a Q^2^ of 0.813 (Figure 3B), indicating that separation observed in the PLS-DA model in Figure 3A did not occur by chance. Again, similar to wild-type control mice, no overlap was observed between clusters of male and female mice when PLS-DA plots were graphed separately for the individual time points at 4, 6, 8, 12, 16, and 20 weeks of age (Figures S 7, S 8, S 9, S 10, S 11, S 12 in File S2).

Analysis to investigate which metabolites were responsible for the separation of IL-10 gene-deficient male and female metabolomic clusters on the PLS-DA plots revealed a total of 19 metabolites that directed the separation of females on the PLS-DA plots and 25 metabolites that directed the separation of males (Tables 3, 4). Eight metabolites were unique to female Il-10 gene-deficient mice at week 4 (fucose, carnitine, urea, isoleucine, adipate, taurine, betaine, and o-acetylcarnitine). Two metabolites were unique to males at week 4 (tyrosine, xylose) and 3 metabolites were unique to males at week 20 (uracil, mannose, and N,N-dimethylglycine). Two metabolites (1-methylnicotinamide and lactate) were dominant in females at each time point. Only one metabolite (succinate) was dominant in males at each time point (Tables 3, 4).

**Table 3:** Gender specific metabolites that contribute to the separation of female IL-10 gene-deficient mice on the PLS-DA plot at different experimental time points.

Metabolites	Age (Weeks)					
	4	6	8	12	16	20
1. Fucose	+					
2. Carnitine	+					
3. Urea	+					
4. Isoleucine	+					
5. Adipate	+					
6. Taurine	+					
7. Betaine	+					
8. 0-Acetylcarnitine	+					
9. 1-Methylnicotinamide	+	+	+	+	+	+
10. Lactate	+	+	+	+	+	+
11. cis-Aconitate	+	+	+			+
12. Pyruvate	+	+		+	+	+
13. Hippurate		+	+			
14. Trimethylamine N-Oxide		+	+	+		+
15. Glutarate		+	+	+	+	+
16. N-Carbamoyl-β-Alanine			+	+		+
17. 3-Methyl-2-Oxovalerate				+		
18. Glycolate					+	
19. Xylose					+	
**Total number of metabolites**	**12**	**7**	**7**	**7**	**6**	**7**

The plus sign (+) in a column indicates a metabolite that contributed to the separation of the respective gender on the PLS-DA plot.

**Table 4:** Gender specific metabolites that contribute to the separation of male IL-10 gene-deficient mice on the PLS-DA plot at different experimental time points.

Metabolites	Age (Weeks)					
	4	6	8	12	16	20
1. Tyrosine	+					
2. Xylose	+					
3. Ethanolamine	+	+				
4. Succinate	+	+	+	+	+	+
5. Asparagine	+		+	+		+
6. 2-oxoisocaproate		+				
7. Betaine		+				
8. Pyro-glutamate		+	+	+		
9. Creatine		+	+		+	+
10. Valine		+	+		+	+
11. Trimethylamine		+	+	+	+	+
12. N-Isovaleroylglycine		+	+	+	+	+
13. Taurine		+	+	+	+	+
14. Methylamine		+	+	+	+	+
15. Isoleucine		+		+		+
16. 2-Aminobutyrate		+			+	
17. Butyrate			+	+	+	
18. 3-Hydroxybutyrate				+		
19. Adipate				+		
20. Malonate				+	+	+
21. Carnitine					+	
22. Glucose					+	
23. Uracil						+
24. Mannose						+
25. N,N-Dimethylglycine						+
**Total number of metabolites**	**5**	**13**	**10**	**12**	**12**	**13**

The plus sign (+) in a column indicates a metabolite that contributed to the separation of the respective gender on the PLS-DA plot.

### Comparison of Wild-type to IL-10 Gene-deficient Mice Metabolomic Profiles

To investigate the impact of IBD on the gender-specific metabolomic profile identified above, we next compared metabolites from IL-10 gene-deficient mice with those from wild-type control mice. While the number of individual metabolites (VIP score >1) that determine the separation of males and females into metabolomic clusters is similar in IL-10 gene-deficient mice and in wild-type mice (19 female and 25 male in IL-10 gene-deficient versus 20 female and 23 male in wild-type), the individual metabolites and their distribution at varying ages differ substantially.

In IL-10 gene-deficient mice, the gender-separating metabolites at week 4 were mainly from metabolites that contributed to the separation of females (i.e., female dominant) (12 out of 19) as opposed to metabolites that contributed to the separation of males (i.e., male dominant) (5 out of 25) (Tables 3, 4). This pattern was in contrast to that seen in wild-type control mice where the gender-separating metabolites at 4 weeks were male dominant metabolites (13 out of 23) relative to female metabolites (6 out of 20) (Tables 1, 2). An opposite pattern emerges when animals at 16 and 20 weeks of age are examined. The majority of gender-separating metabolites at these ages are female dominant in wild-type mice (17/20 and 15/20 at weeks 16 and 20, respectively for females compared to 7/23 and 9/23, respectively for males) (Tables 1, 2); but male dominant in Il-10 gene-deficient mice (12/25 and 13/25 at weeks 16 and 20, respectively for males compared to 6/19 and 7/19, respectively for females) (Tables 3, 4).

A further comparison of the gender-separating metabolites in IL-10 gene-deficient and wild-type control mice revealed metabolites that overlapped as well as metabolites that were unique for gender separation across IL-10 gene-deficient and wild-type mouse background (Table 5). Basically, the metabolites that contribute to the separation of male from female can be divided into four groups: a) those that are shared between IL-10 gene-deficient and wild-type mice at one or more time points, b) those that are common in IL-10 gene-deficient and wild-type mice but never contribute to the gender separation at the same time point, c) those that are unique for wild-type gender separation, and d) those that are unique for IL-10 gene-deficient gender separation (Table 5).

**Table 5:** Metabolites that contribute to the separation of male from female for wild-type control (a) and IL-10 gene-deficient mice (b).

Metabolites thatcontribute toseparation of males onthe PLS-DA plot	Age (Weeks)						Metabolites thatcontribute to separationof females on the PLS-DAplot	Age (Weeks)					
	4	6	8	12	16	20		4	6	8	12	16	20
**Metabolites Common to Wild-Type (a) And IL-10 Gene-Deficient (b) Mice**													
Succinate	ab	ab	ab	ab	ab	ab	1-Methylnicotinamide	ab	ab	ab	ab	ab	ab
Taurine		ab	ab	ab	ab	ab	Lactate	b	ab	ab	ab	ab	ab
Valine		ab	ab	ab	ab	ab	Trimethylamine N-oxide	b	ab	ab	ab	b	ab
Trimethylamine	a	ab	ab	ab	ab	ab	Pyruvate	b	ab	ab	b	ab	ab
Methylamine	a	ab	ab	ab	ab	ab	Glutarate		ab	ab	ab	ab	B
N-Isovaleroylglycine	a	ab	ab	ab	ab	ab	N-Carbomoyl-β-alanine			ab	ab	a	ab
Ethanolamine	ab	ab		a	a		3-Methyl-2-Oxovalerate	a	a	a	ab	a	a
Butyrate		a	ab	ab	b	a	Glycolate		a	a		ab	a
Creatine		ab	ab		b	b	cis-Aconitate	b	b	b		a	ab
Isoleucine	a	ab	a	b		b							
Adipate	a	a	a	ab									
Pyroglutamate		b	b	ab									
3-Hydroxybutyrate				ab									
**Metabolites Common But At Different Time Points In Wild-Type (a) And IL-10 Gene-Deficient (b) Mice**													
N,N-Dimethylglycine	a	a	a			b	Fucose	b			a	a	a
Uracil	a					b	Betaine	b				a	
2-Aminobutyrate		b			b	a							
**Metabolites Unique To Wild-Type (a) Mice**													
Trigonelline	a						Guanidoacetate	a				a	
Dimethylamine	a						Formate	a	a	a		a	
2-Hydroxyisobutyrate	a	a	a				Glycin	a					a
citrate	a			a			2-Oxoisocaproate			a	a	a	a
trans-Aconitate				a			Acetate				a	a	
Methanol				a			Allantoin					a	a
Hippurate						a	Mannose					a	a
							Phenylacetylglycine						a
							3-Indoxysulfate						a
**Metabolites Unique To IL-10 Gene-Deficient (b) Mice**													
Tyrosine	b						Urea	b					
Xylose	b						O-Acetylcarnitine	b					
Asparagine	b		b	b		b	Taurine	b					
2-Oxoisocaproate		b					Isoleucine	b					
Betaine		b					Adipate	b					
Malonate				b	b	b	Carnitine	b					
Carnitine					b		Hippurate		b	b			
Glucose					b		Xylose					b	
Mannose						b							
**Total Common**	**2**	**9**	**8**	**10**	**6**	**6**	**Total Common**	**1**	**5**	**6**	**6**	**5**	**6**
**Total Wild-Type**	**11**	**4**	**4**	**4**	**1**	**3**	**Total Wild-Type**	**4**	**3**	**4**	**3**	**11**	**9**
**Total IL-10 Gene-Deficient**	**3**	**4**	**2**	**3**	**6**	**7**	**Total IL-10 Gene-Deficient**	**12**	**2**	**2**	**1**	**2**	**1**

Metabolites that separate gender and are equally present in the IL-10 gene-deficient and wild-type control mice are presumably less affected by the underlying genetic difference in the two mouse strains that ultimately leads to the development of IBD. This group of metabolites include: succinate, taurine, valine, trimethylamine, methylamine, and N-isovaleroylglycine for male-dominant metabolites and 1-methylnicotinamide, lactate, trimethylamine-N-oxide, pyruvate, and glutarate for female-dominant metabolites (Table 5). Conversely, metabolites that separate gender but are unique to the wild-type control and IL-10 gene-deficient mice are presumably more affected by the development of intestinal inflammation.

### The Effect of age on Metabolomic Profiles

**Figure 4:**
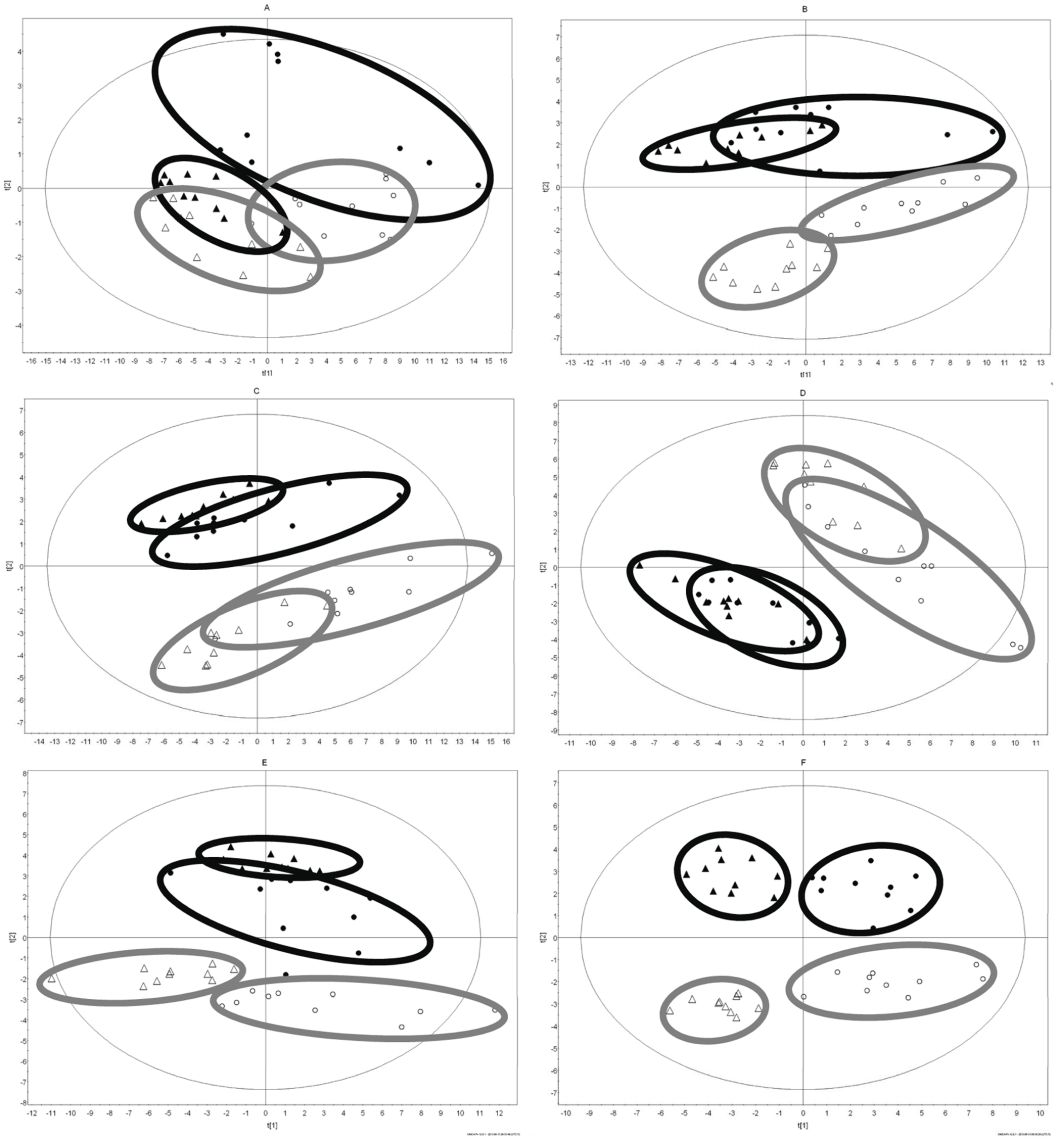
Comparison of metabolomic profiles between male and female IL-10 gene-deficient and wild-type mice. Metabolomic analysis comparing groups of female wild-type (open triangle), male wild-type (closed triangle), female IL-10 gene-deficient (open circles), and male IL-10 gene-deficient (closed circles) mice. PLS-DA analysis from 10 mice per group are shown at weeks 4 (A), 6 (B), 8 (C), 12 (D), 16 (E), and 20 (F).

Finally, having shown that the metabolomic profile can separate males from females in both IL-10 gene-deficient and wild-type control mice and that the individual metabolites responsible for this separation are affected by both the genetics of the mice (i.e., IL-10 gene deficiency) and the development of intestinal inflammation, we next examined the PLS-DA plots at each age (Figure 4). At all 6 age points tested, metabolomic profiles from wild-type males and females form tighter clusters than the corresponding profiles of male and female IL-10 gene-deficient mice.

Examining metabolomic separation of gender, at week 4 there is an overlap of metabolomic profiles between male and female IL-10 and between male and female wild-type (Figure 4A), consistent with the low numbers of common gender separating metabolites at this time point (Table 5). However, by week 6 and beyond (Figures 4B–F), separation of the genders is complete and pronounced, reflecting the higher number of common gender separating metabolites (Table 5).

In contrast to the results for gender, examining the intestinally inflamed IL-10 gene-deficient versus wild-type control mice, there is overlap of the metabolomic profiles until weeks 16 and 20, the time of maximal intestinal inflammation (Figures 4E, 4F). This difference suggests that a significant effect is exerted by the metabolites emanating as a consequence of the inflammation.

## Discussion

This study demonstrates that the metabolomic profile of male and female gender is manifested in unique metabolites and that these profiles undergo gender-specific changes during the development of intestinal inflammation.

Using urine-based NMR methodology combined with multivariate analysis we demonstrated that healthy wild-type control male and female mice form distinct metabolomic clusters. A kinetic analysis of the major metabolites involved in this gender separation further revealed a change in the metabolomic profile as the animals aged, up to 20 weeks. Similarly, the metabolomic profiles of male and female IL-10 gene-deficient mice (an experimental model of IBD) separated into distinct clusters according to gender. Nevertheless, the metabolites leading to these gender-directed metabolomic profiles in the IL-10 gene-deficient mice differ from those identified for the wild-type control mice, even at an age when there is no gastrointestinal inflammation (i.e., 4, 6 and 8 weeks) [39], implying an important role of genetics to the metabolomic profile. This is further supported by the differences observed in previous findings demonstrating increased pro-inflammatory cytokine release in young IL-10 gene-deficient mice before identifiable histopathologic injury [39].

In order to confirm disease states for each group of mice, colons were excised and histology was performed on each mouse at the end of the urine collection time periods (wk 20). Histology scores ranged from 0 (no injury) to 10 (maximal injury). Histology scores for the IL-10 gene-deficient mice were higher than those found at base level for the wild-type mice (Figure 2A) regardless of gender.

A comparative analysis of the metabolites responsible for the different metabolomic profiles in IL-10 gene-deficient and wild-type control mice identified common metabolites that are shared between the two strains and, therefore, appear to contribute to gender differentiation independent of IBD or the IL-10 gene deficiency. Metabolites that separate male gender in both IL-10 gene-deficient and wild-type mice include the amino acids valine and taurine, succinate (a component of the citric acid cycle), as well as methylamine, trimethylamine, and N-isovaleroylglycine (a component of fatty acid metabolism). Metabolites that separate female gender in both IL-10 gene-deficient and wild-type mice include primarily oxidation products of metabolic intermediates such as lactate, pyruvate, glutarate, and trimethylamine-N-oxide in addition to 1-methylnicotinamide.

Unlike the common metabolites that determine gender separation over a wide range of ages, we have identified unique metabolites that play a role in gender separation at one or only a few selected ages in relation to the development of IBD and gastrointestinal inflammation. We propose that these unique metabolites are candidate biomarkers for the development of intestinal inflammation. Unique metabolites are not universally identified at each age point and are frequently present in both females and males, particularly those involved in gender separation of IL-10 gene-deficient mice. For example carnitine, an amino acid with a major role in fatty acid metabolism, is a female-dominant metabolite in IL-10 gene-deficient mice at week 4 before the histopathologic onset of disease and a male dominant metabolite at week 16 when IBD is established in these mice [11]. Hippurate, a metabolite that has been previously associated with intestinal inflammation in humans, appears as a male-dominant metabolite at week 20 in wild-type mice and as a female-dominant metabolite at the start of pathophysiological inflammation at weeks 6 and 8 in IL-10 gene-deficient mice [9], [41]. Mannose, a carbohydrate involved in cellular recognition processes [42], is involved in gender-separation as a male-dominant metabolite at week 20 in IL-10 gene-deficient mice with IBD and as a female-dominant metabolite at the same time point in wild-type mice. These unique metabolites may also play a role in the observed differences in disease severity observed between male and female mice in the IL-10 gene-deficient group. This is consistent with the analysis by Mittelstrass et al. which also suggested that gender can lead to gender specific metabotypes [43].

In comparing the metabolites responsible for the metabolomic profile of gender in healthy IL-10 gene-deficient and wild-type control mice over the time frame of IBD development, our study revealed a different pattern in clustering between males and females depending on the pattern of common versus unique metabolites among male- and female-dominant compounds. In this regard, cluster separation is weak at week 4 with very few common metabolites (Figure 4A, Table 5). An increase in metabolites that are common for gender differentiation in both strains leads to a separation of male and female clusters by week 6 (Figure 4B).

Over the time frame the intestinal inflammation develops, from 8 to 16 weeks, there are more common metabolites that define male gender in both IL-10 gene-deficient and wild-type controls mice than common metabolites that define female gender. Thus it appears that for males, gender influences the metabolites to a greater extent than does the animal’s genetic profile or the intestinal inflammation. In contrast, the lower number of common metabolites that define female gender suggests that in females, the animal’s genetic profile and intestinal inflammation plays a larger role in the metabolomic profile.

In this study, using urine-based NMR technology, we demonstrated that metabolomic profiles, in a genetically-induced model of IBD, are gender, age and disease specific. Comparison of metabolite patterns computed from the analysis of male and female, and IL-10 gene-deficient and wild-type control mice, revealed dependent and independent changes in metabolites that result in distinctive metabolomic profiles that are then further impacted by the development of intestinal inflammation. The knowledge of metabolomic profile differences by gender and by disease states has potential clinical implications in the design of both biomarkers of disease as well as the development of optimal therapies.

## Supporting Information

File S1 **Figures S 1–S 6.** Partial least squares-discriminant analysis (PLS-DA) of male (closed triangle) versus female (open triangle) wild-type mice at each individual time point tested (weeks 4, 6, 8, 12, 16, and 20) demonstrate a gender separation in two distinct clusters on each plot.
(TIFF)

File S2 **Figures S 7–S 12.** Partial least squares-discriminant analysis (PLS-DA) of male (closed circle) versus female (open circle) IL-10 gene-deficient mice at each individual time point tested (weeks 4, 6, 8, 12, 16, and 20) demonstrate a gender separation in two distinct clusters on each plot.
(TIFF)
