# Supplementary figures and images for: Metabolomic Profiles Are Gender, Disease and Time Specific in the Interleukin-10 Gene-Deficient Mouse Model of Inflammatory Bowel Disease
Source: PLoS One. 2013 Jul 9;8(7):e67654. doi: 10.1371/journal.pone.0067654 (PMC3706546; doi:10.1371/journal.pone.0067654)

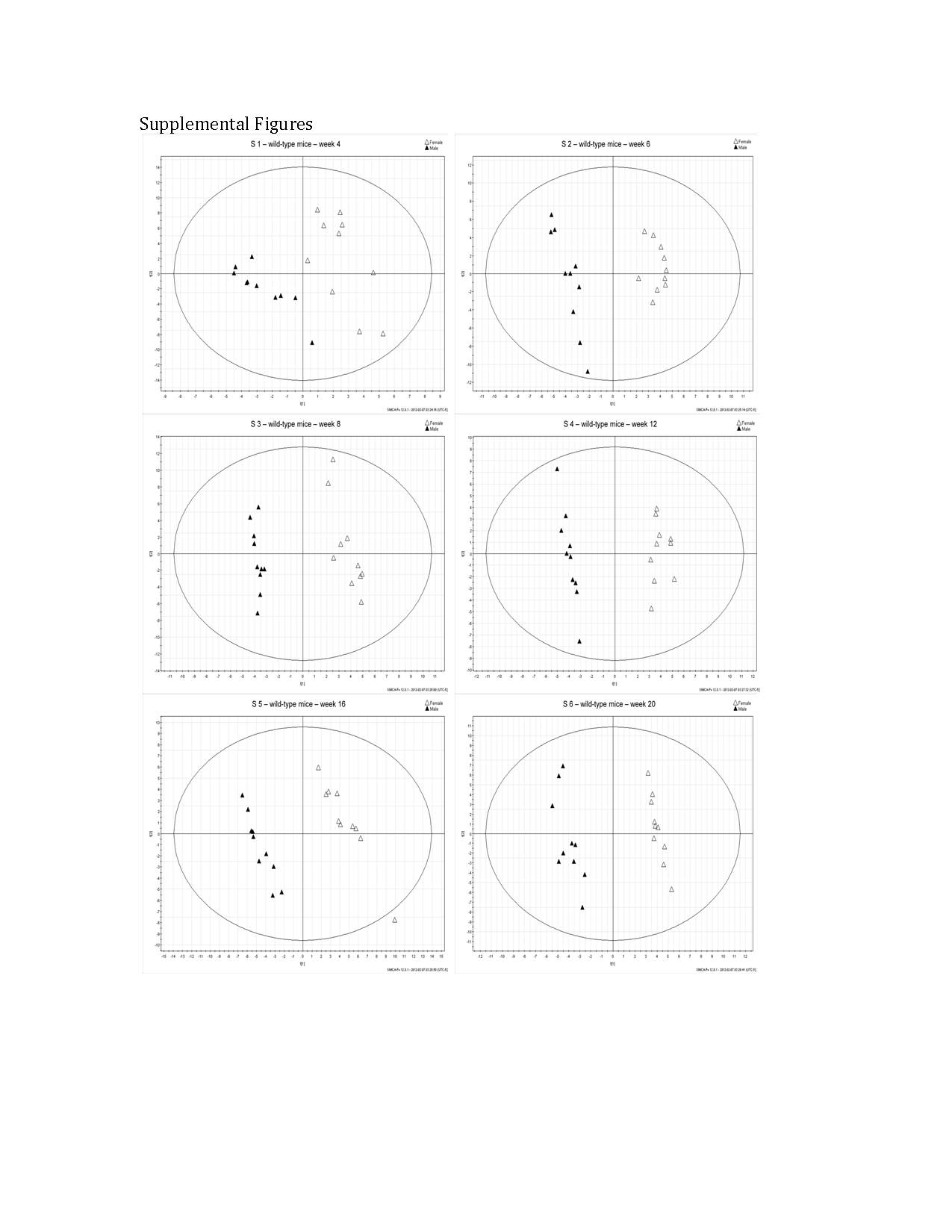

Supplement: File S1 — Figures S 1–S 6. Partial least squares-discriminant analysis (PLS-DA) of male (closed triangle) versus female (open triangle) wild-type mice at each individual time point tested (weeks 4, 6, 8, 12, 16, and 20) demonstrate a gender separation in two distinct clusters on each plot. (TIFF) [file pone.0067654.s001.tiff]

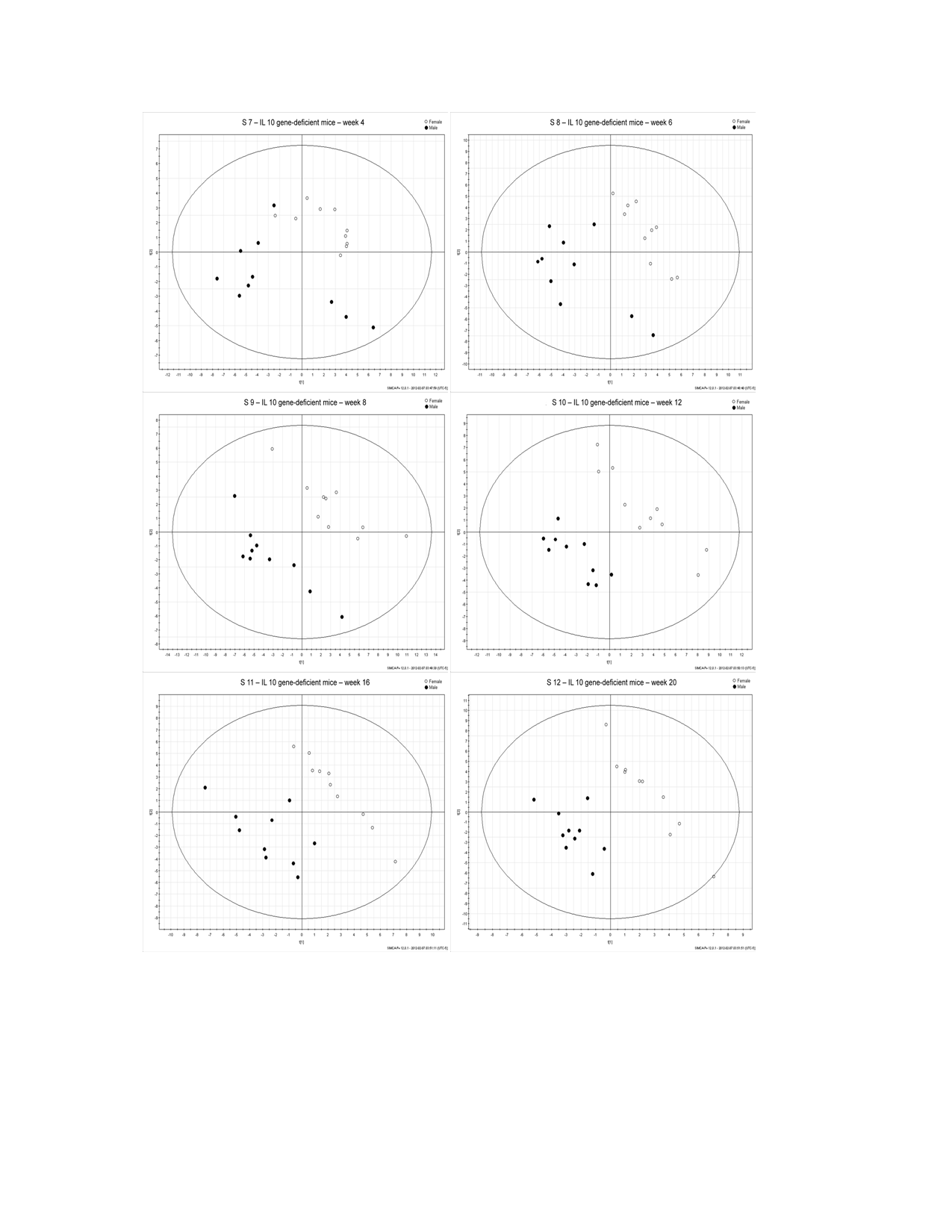

Supplement: File S2 — Figures S 7–S 12. Partial least squares-discriminant analysis (PLS-DA) of male (closed circle) versus female (open circle) IL-10 gene-deficient mice at each individual time point tested (weeks 4, 6, 8, 12, 16, and 20) demonstrate a gender separation in two distinct clusters on each plot. (TIFF) [file pone.0067654.s002.tiff]
